# The effects of two cytotoxic gold(i) carbene compounds on the metabolism of A2780 ovarian cancer cells: mechanistic inferences through NMR analysis†

**DOI:** 10.1039/d3ra04032a

**Published:** 2023-07-19

**Authors:** Veronica Ghini, Michele Mannelli, Lara Massai, Andrea Geri, Stefano Zineddu, Tania Gamberi, Luigi Messori, Paola Turano

**Affiliations:** a Department of Chemistry “Ugo Schiff”, University of Florence Sesto Fiorentino 50019 Italy luigi.messori@unifi.it paola.turano@unifi.it; b Magnetic Resonance Center (CERM), University of Florence Sesto Fiorentino 50019 Italy; c Department of Experimental and Clinical Biomedical Sciences “Mario Serio”, University of Florence Florence 50134 Italy; d Consorzio Interuniversitario Risonanze Magnetiche di Metallo Proteine (CIRMMP) Sesto Fiorentino 50019 Italy

## Abstract

NMR metabolomics is a powerful tool to characterise the changes in cancer cell metabolism elicited by anticancer drugs. Here, the large metabolic alterations produced by two cytotoxic gold carbene compounds in A2780 ovarian cancer cells are described and discussed in comparison to auranofin, in the frame of the available mechanistic knowledge.

## Abstract 2

The changes in the exo- and endo-metabolome of A2780 ovarian cancer cells caused by two cytotoxic gold carbene compounds were monitored *via*^1^H NMR.

## Graphical abstract

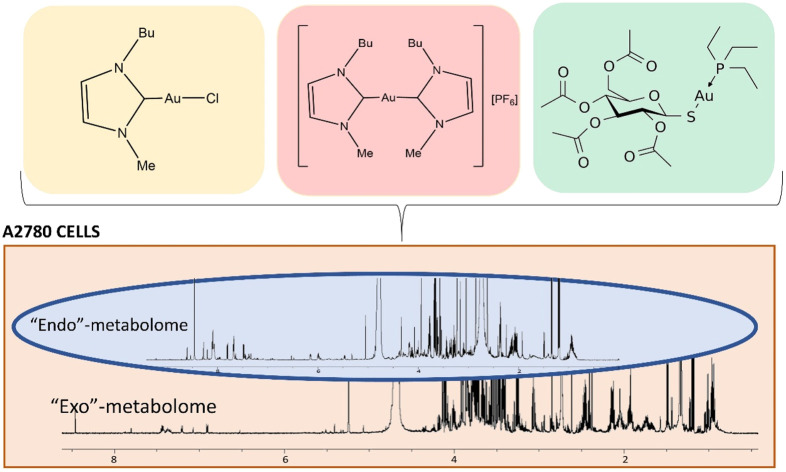

Gold compounds are drawing increasing attention as a novel class of experimental anticancer agents.^1^ Indeed, a large variety of structurally diverse gold compounds have been prepared and evaluated in recent years and have manifested promising anticancer properties both *in vitro* and *in vivo*.^1^ Auranofin (AF), an FDA approved antiarthritic gold drug, endowed with remarkable antiproliferative properties, has become the reference compound for this class of anticancer agents.^2^ Two gold(i) carbene complexes, *i.e.*, Au(NHC)Cl and [Au(NHC)_2_]PF_6_, where NHC is a 1-butyl-3-methyl-imidazide-2-ylidene ligand, have been synthesized and characterized in our laboratory a few years ago.^3,4^ Notably, this N-heterocyclic carbene is a very strong ligand for gold(i) as it possesses donor properties similar to phosphines, affording a very stable gold(i) coordination; the resulting gold(i) complexes show potent cytotoxic properties toward A2780 ovarian cancer cells. The chemical structures of Au(NHC)Cl and [Au(NHC)_2_]PF_6_, are reported in Fig. 1; the structure of auranofin is also shown for comparison. In both compounds, the gold(i) center linearly coordinates a NHC ligand and, as second ligand, a chloride in the mono-carbene complex, Au(NHC)Cl, or another N-heterocyclic carbene in the bis-carbene complex, [Au(NHC)_2_]PF_6_. Owing to the presence of chloride, a relatively weak gold(i) ligand, in the mono-carbene complex, the two compounds are deeply distinct from the chemical point of view: Au(NHC)Cl is neutral, less stable and more reactive while [Au(NHC)_2_]PF_6_ is mono-cationic, highly stable in biological fluids and far less reactive.^5^

**Fig. 1:**
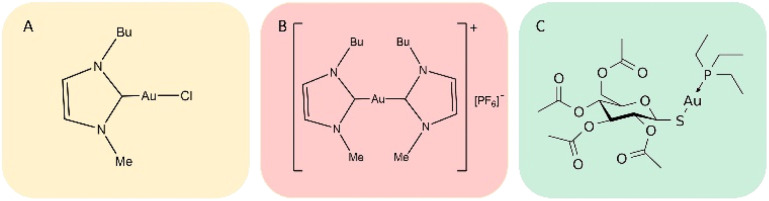
The chemical structures of (A) Au(NHC)Cl; (B) [Au(NHC)_2_]PF_6_; (C) auranofin.

Many investigations have been carried out so far to disclose the actual modes of action of anticancer gold(i) compounds. A widely accepted mechanistic hypothesis for AF postulates that the large increase in intracellular oxidative stress observed upon treatment is the consequence of strong inhibition of the selenoenzyme thioredoxin reductase (TrxR); in turn, such severe oxidative stress causes a profound mitochondrial dysfunction ultimately leading to apoptosis.^6–8^ Yet, some additional targets including NFKB2 and CHORDC1 were disclosed for AF by recent proteomic studies suggesting that AF may possess a more complex, multitarget mechanism.^9^

The mechanistic studies previously conducted in our laboratory on Au(NHC)Cl and [Au(NHC)_2_]PF_6_, beyond documenting a similar and potent inhibition of the selenoenzyme TrxR, revealed the occurrence of additional distinctive features still to be fully understood.^4^ Interestingly, [Au(NHC)_2_]PF_6_ turned out to be far more cytotoxic than Au(NHC)Cl (and AF itself) by at least a factor 10. Through an integrated approach involving biochemical studies, proteomic and redox-proteomic analyses we demonstrated that both gold carbenes trigger the oxidation of proteins belonging to two main functional categories: carbohydrate metabolism, and cytoskeleton organization/cell adhesion; moreover, the occurrence of a severe mitochondrial dysfunction and the conversion to a more glycolytic phenotype were clearly documented in both cases, with [Au(NHC)_2_]PF_6_ being again far more effective than Au(NHC)Cl in producing such effects.^4,10^

NMR metabolomics is a very informative research tool capable of revealing the main metabolic alterations caused by a variety of drugs in cancer cells.^11,12^ Recently, we have implemented this investigative approach to characterise the metabolic perturbations induced by a few metallodrugs in a reference cancer cell line.^13,14^

We addressed the cellular alterations caused by AF in A2780 ovarian cancer cells revealing a large increase of intracellular glutathione concentration as the main effect of the treatment;^13^ in line with these findings, a subsequent proteomic analysis has shown that AF treatment in A2780 cells induces the overexpression of two enzymes involved in GSH biosynthesis, namely GCLC (glutamate-cysteine ligase catalytic subunit) and GCLM (glutamate-cysteine ligase regulatory subunit).^8^ Additionally, some significant effects on the glucose metabolism produced by AF treatment were observed.^13^

Here, ^1^H NMR metabolomics is used to analyze the metabolic changes brought about by the treatment of A2780 cancer cells with either Au(NHC)Cl or [Au(NHC)_2_]PF_6_. Results are compared with those previously obtained for AF.^13^ The experiments were carried out according to the experimental protocol previously established. Briefly, A2780 cells were treated for 6, 12 and 24 h with a concentration of the Au(i) drugs equal to their respective IC_50_ values at 72 h (Table S1†) to work under equitoxic conditions and monitor the early drug-induced metabolic changes before the occurrence of significant apoptosis. The NMR analysis was performed both on the cellular endo- and exo-metabolome (*i.e.* the cell lysates and the growth media). Typical NMR spectra of cell lysates and growth media of untreated cells and cells treated with the two gold compounds – recorded after 24 h – are shown in Fig. S1–S3.†

Significant spectral alterations were detected in response to these treatments. Pairwise comparisons between A2780 control cells and Au(NHC)Cl- or [Au(NHC)_2_]PF_6_-treated cells revealed that the number of altered metabolites was higher between controls and [Au(NHC)_2_]PF_6_-treated cells, with 13 metabolites altered in cell lysates and 22 in the growth media. Among these, 8 metabolites for lysates and 14 metabolites for growth media were also found to be significantly different between control and Au(NHC)Cl groups showing the same, but less marked, trend.

Detailed analysis of the NMR data permits to define the quantitative changes elicited by the gold drugs in the endo- and exo-metabolomics profiles. Results at 24 h are synoptically shown in Fig. 2 and 3. Similar comparisons are provided in Fig. S4 and S5† for the shorter treatment times.

**Fig. 2:**
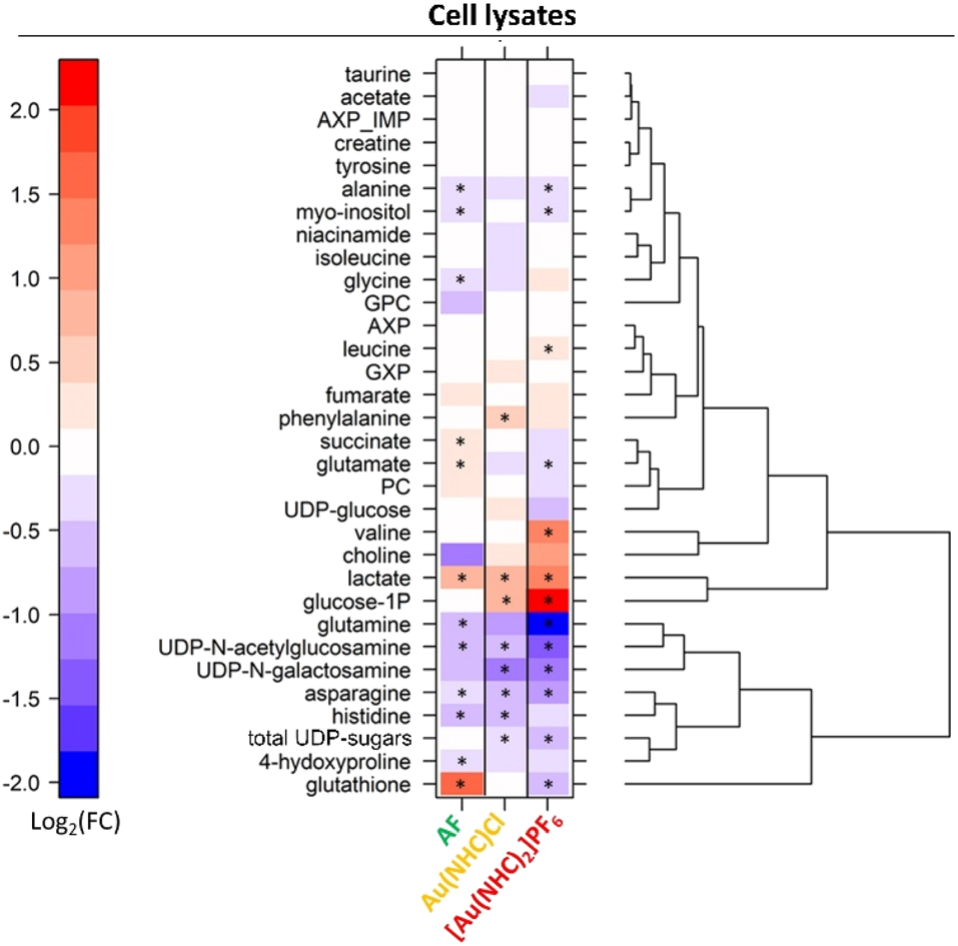
Level plot of fold changes of the intracellular metabolites (cell lysates) upon 24 h of treatment of A2780 cells with AF, Au(NHC)Cl and [Au(NHC)_2_]PF_6_. Red/blue colours indicate the higher/lower metabolite concentration in Au(i) drug-treated cells with respect to control samples (log_2_(FC)). The brightness of the colours corresponds to the magnitude of FC. Asterisks indicate statistical significance (*p*-value < 0.05).

**Fig. 3:**
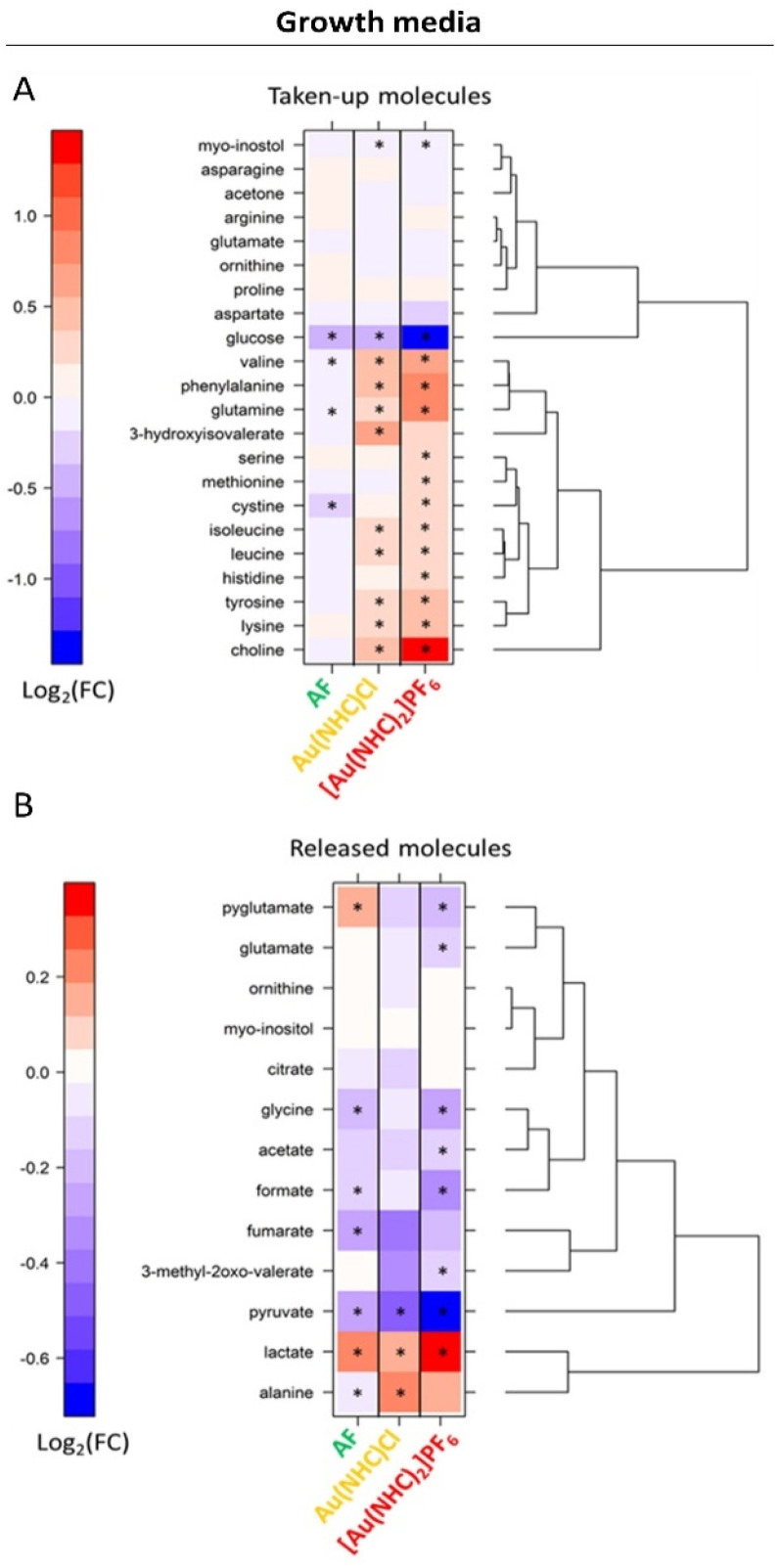
Level plots of fold changes of the extracellular metabolites (growth media) upon 24 h of treatment of A2780 cells with AF, Au(NHC)Cl and [Au(NHC)_2_]PF_6_. Red/blue colours indicate the higher/lower metabolite concentration in Au(i) drug-treated cells with respect to control samples (log_2_(FC)). The metabolites are divided into two different classes, *i.e.* those that are taken up from the medium (A) and those that are released into the medium (B). While for the molecules that are released, lower concentration levels upon treatment mean a lower release, for the molecules that are taken up from the starting media, lower levels upon treatment mean a greater consumption of nutrients, *i.e.* increased uptake, and *vice versa*. The brightness of the colours corresponds to the magnitude of FC. Asterisks indicate statistical significance (*p*-value < 0.05).

Upon inspection of the endo-metabolome data, the following observations can be made:

- Au(NHC)Cl and [Au(NHC)_2_]PF_6_ do not induce any increase in GSH; [Au(NHC)_2_]PF_6_ rather causes a decrease of GSH while Au(NHC)Cl does not affect it significantly.

- Both gold carbenes induce a significant increase in intracellular lactate. The effect is far larger in the case of [Au(NHC)_2_]PF_6_.

- A relatively large increase of glucose-1-phosphate is detected for both Au(NHC)Cl and [Au(NHC)_2_]PF_6_, consistent with the greater uptake of glucose.

- [Au(NHC)_2_]PF_6_ produces an evident increase in the concentration of Val and a significant decrease in the concentrations of Gln, Asn, Glu, Ala, UDP *N*-galactosamine and UDP *N*-acetylglucosamine. On the whole, Au(NHC)Cl produces qualitatively similar but less intense changes in these metabolites; Au(NHC)Cl does not affect at all Val concentration.

Of interest is also the analysis of the effects produced by Au(NHC)Cl and [Au(NHC)_2_]PF_6_ on culture media. Data for 24 h treatment are shown in Fig. 3; results at shorter times are reported in Fig. S5.† The comparative analysis of these results leads to the following observations:

- The uptake of glucose is greatly enhanced by [Au(NHC)_2_]PF_6_ and moderately enhanced by Au(NHC)Cl and AF;

- The release of lactate is greatly enhanced by [Au(NHC)_2_]PF_6_ and moderately enhanced by Au(NHC)Cl and AF;

- The uptake of several amino acids from the growth medium such as Val, Ile, Leu, Phe, Gln, Tyr and Lys along with the uptake of choline are generally decreased by [Au(NHC)_2_]PF_6_ and Au(NHC)Cl;

- The treatment with [Au(NHC)_2_]PF_6_ strongly decreases the release of pyruvate, formate and Gly. This effect is still present but less pronounced in the case of Au(NHC)Cl and AF.

Thus, NMR metabolomics reveals that Au(NHC)Cl and [Au(NHC)_2_]PF_6_ produce, on the whole, a number of relevant alterations in A2780 cells metabolism that are quite different from those caused by AF (Fig. S6†). These results can be tentatively interpreted in the frame of the current mechanistic knowledge existing on [Au(NHC)_2_]PF_6_, Au(NHC)Cl and AF, as detailed below.

The mechanisms of Au(NHC)Cl and [Au(NHC)_2_]PF_6_ are less explored and less known than those of AF. However, a recent proteomic study of ours tried to delineate the main mechanistic features of these two compounds.^4^ It emerged that the two gold carbene compounds are highly cytotoxic with [Au(NHC)_2_]PF_6_ being far more potent than Au(NHC)Cl. Similarly to AF, both gold carbenes are able to strongly inhibit TrxR and to induce severe mitochondrial damage. Yet, the conversion to a glycolytic phenotype is far more evident here than in the case of AF, especially in the case of [Au(NHC)_2_]PF_6_.^4^

NMR metabolomics has turned out to be a very appropriate tool to dissect these effects at the molecular level; indeed, a greater uptake of glucose and a greater production of lactate are clearly documented here, starting from the very early times of treatment. On the other hand, NMR results show that both gold carbenes – at variance with AF – are not able to stimulate GSH production, which in AF can be attributed to the overexpression of the two enzymes GCLC and GCLM, involved in its biosynthesis. Rather, in the case of [Au(NHC)_2_]PF_6_, a small but significant depletion of GSH is detected.

Both gold carbenes also induce some significant alteration in the intra- and-extracellular concentrations of several amino acids. More in detail, the intracellular concentrations of the amino acids Gln and Asn are decreased, as in the case of AF. Instead, at variance with AF, the extracellular concentrations of several amino acids, including Gln, Val, Ile, Leu, Phe, His, Tyr, Lys, *etc.* are increased, suggesting a reduction of their cellular uptake. These changes are evocative of a defective mitochondrial metabolism.^15^

Moreover, in line with AF, both gold carbenes induce a significant reduction of the UDP sugars (UDP-acetyl-glucosamine and UDP-galactosamine). Interestingly, a significant decrement of these molecules was also observed in A2780 cells upon platinum drugs treatment^14^ and was related to a reduction of cancer aggressiveness^16^ and ER stress.^17^

In conclusion, NMR metabolomics has revealed that these gold carbene compounds induce relevant and specific alterations in the cancer cell metabolome that are significantly different from those induced by AF. The main metabolomic changes observed for each gold(i) compound are summarised in Fig. 4. It is evident that both gold carbenes cause a relevant shift of the cell metabolism toward a glycolytic phenotype. This shift is well documented by the increase in lactate production that is particularly pronounced in the case of [Au(NHC)_2_]PF_6_. It is very likely that conversion to a glycolytic phenotype is the direct consequence of potent inhibition of the mitochondrial function. Indeed, in a previous study, we reported that both Au(NHC)Cl and [Au(NHC)_2_]PF_6_ produce a net reduction in oxygen consumption as the result of their anti-mitochondrial actions. In the case of auranofin all these effects are also observed but to a smaller degree.

**Fig. 4:**
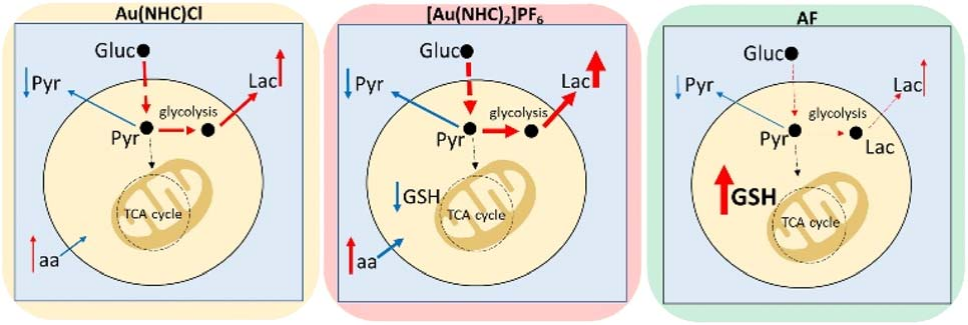
Outline of the main alterations observed *via* NMR-metabolomics upon treatment of A2780 cells with Au(NHC)Cl, [Au(NHC)_2_]PF_6_ and AF; red upregulated, blue downregulated pathways; the thickness of the arrowed lines is proportional to the magnitude of the observed changes. Gluc = glucose; Pyr = pyruvate; Lac = Lactate; aa = Gln, Val, Ile, Leu, Phe, Tyr, His, Lys.

Though these gold carbene compounds share a few crucial mechanistic features of AF, such as a strong inhibition of the selenoenzyme TrxR associated to induction of oxidative stress, it is evident that other aspects of their mode of action are deeply distinct leading to a greater mitochondrial damage and to a stronger conversion to a glycolytic phenotype. Overall, these mechanistic differences result in a different and greater impact on the metabolome of A2780 cancer cells.

Notably, from the present study, it clearly emerges that the nature of the ligands coordinated to the gold(i) center crucially modulates its biological actions by tuning its affinity toward different protein targets. This is particularly true for the strong carbene ligands that are not easily removed from direct gold(i) coordination and stay coordinated to the metal for a long time, even within the cellular environment. In the case of the biscarbene gold complex, retention of the two carbene ligands in the biological media even results in long term conservation of the monopositive charge conferring to this complex the property of a delocalised lipophilic cation (DLC) with a specific tropism for mitochondria. These considerations open the stage for further SAR studies on similar anticancer gold(i) compounds.

Also, in view of the here reported results, we can suggest that the large dysregulation of cellular metabolism produced by these gold carbene compounds is the real cause of cancer cell death, in line with literature.^18,19^ It follows that the behaviour of these cytotoxic gold compounds largely differs from established anticancer platinum(ii) drugs that were earlier reported to produce relatively modest metabolic alterations in the same cancer cell line, possibly due to their prevalent DNA damaging mechanism.^14^ Finally, this study further demonstrates that NMR metabolomics is a powerful and effective tool to disclose the metabolic effects of anticancer metallodrugs offering the chance to establish valuable correlations between the main mechanistic aspects and the observed metabolic alterations.

## Author contributions

Conceptualization, L. M. and P. T.; methodology, V. G., P. T., L. M. and T. G.; formal analysis, V. G., S. Z.; investigation, V. G., S. Z., M. M., and T. G.; resources, La. M. and A. G.; writing – original draft, L. M., P. T., V. G., La. M.; writing–review & editing, S. Z., A. G., M. M., and T. G.; supervision, L. M. and P. T.; funding acquisition, L. M. and P. T. S. Z. contributed to this work before the start of his PhD.

## Conflicts of interest

There are no conflicts to declare.

## Supplementary Material

RA-013-D3RA04032A-s001
