# Supplementary material for: The effects of two cytotoxic gold(i) carbene compounds on the metabolism of A2780 ovarian cancer cells: mechanistic inferences through NMR analysis
Source: RSC Adv. 2023 Jul 19;13(31):21629–32. doi: 10.1039/d3ra04032a (PMC10354608; doi:10.1039/d3ra04032a)
Supplement: RA-013-D3RA04032A-s001 [file RA-013-D3RA04032A-s001.pdf]

Electronic Supplementary Material (ESI) for RSC Advances.
This journal is © The Royal Society of Chemistry 2023

The effects of two cytotoxic gold(I) carbene compounds on the metabolism of A2780 ovarian cancer cells: mechanistic inferences through NMR analysis.

Veronica Ghini[a,b], Michele Mannelli[c], Lara Massai[a], Andrea Geri[a], Stefano Zineddu[a,b], Tania Gamberi[c], Luigi Messori[a*] and Paola Turano[a,b,d*]

[a.] *Department of Chemistry "Ugo Schiff", University of Florence, Sesto Fiorentino 50019, Italy. E-mail: luigi.messori@unifi.it; paola.turano@unifi.it;*
[b.] *Magnetic Resonance Center (CERM), University of Florence, Sesto Fiorentino 50019, Italy.*
[c.] *Department of Experimental and Clinical Biomedical Sciences "Mario Serio", University of Florence, Florence 50134, Italy.*
[d.] *Consorzio Interuniversitario Risonanze Magnetiche di Metallo Proteine (CIRMMP), Sesto Fiorentino 50019, Italy.*
* Corresponding Authors

Electronic Supporting Information

Table of Contents:

1. Materials and methods Pg. 2
1.1. Gold(I) compounds 2
1.2. Cell culture 2
1.3. Cytotoxic effect on the A2780 cell line 2
1.4. ^{1}H NMR metabolomics 2
1.5. Data analysis 4
2. Results 5
3. Supplementary Tables 7
Table S1 7
4. Supplementary Figures 8
Figure S1 8
Figure S2 9
Figure S3 10
Figure S4 11
Figure S5 12
Figure S6 13

1. Materials and methods

1.1. Gold(I) compounds

- AF. Auranofin was purchased from Sigma-Aldrich with the following characteristics: Code number: A6733, Purity ≥98% (HPLC).

- Au(NHC)Cl and $[Au(NHC)_2]PF_6$ were previously prepared and characterized as described in ref 1.; their purity and homogeneity were again checked prior to use by elemental analysis and ^1H NMR analysis.

1.2. Cell culture

The A2780 human ovarian cancer cell line, purchased from Creative Bioarray (NY 11967, USA) (Lot Nº CSC-C9491J), was used as in vitro model of ovarian cancer. Cells were maintained in RPMI1640 (Euroclone, Milan, Italy) medium supplemented with 10% FBS, 1% glutamine and 1% antibiotics at 37ºC and sub-cultured twice weekly. Split 1:5 (3–6 × 104 cells per cm^2).

1.3. Cytotoxic effect on the A2780 cell line

The inhibition of cell proliferation by AF, Au(NHC)Cl and $[Au(NHC)_2]PF_6$ on the A2780 cell line was evaluated through the MTT (3-(4,5-dimethylthiazol-2-yl)-2,5-diphenyltetrazolium bromide) assay at 72 h of treatment. Briefly, exponentially growing cells were seeded in 96 well-microplates at a density of $5x10^4$/ mL and after 24 h AF, Au(NHC)Cl and $[Au(NHC)_2]PF_6$ were added in fresh RPMI medium at concentrations ranging from 0.0007 to 25 µM and incubated for 72 h. On the day of the assay, cells were treated with 0.5 mg/mL MTT for 1 h at 37 °C. Following precipitation, blue formazan was dissolved in DMSO, and the optical density was measured in a microplate reader interfaced with the Microplate Manager/PV version 4.0 software (BioRad Laboratories, Hercules, USA) at 595 nm. From the absorbance measurements, the half-maximal inhibitory concentration (IC_{50}) value of each compound on A2780 cells was calculated using the GraphPad Prism software version 6.0 (Graphpad Holdings, LLC, USA). All the treatments were performed on two different sets of triplicates. The values are reported in the Table S1.

1.4. ^1H NMR Metabolomics

Sample preparation

A2780 cells were seeded in 60 cm^2 tissue-culture plates at 2.5 × 10^5 cells/ mL (total volume 8 mL) and incubated for 6, 12 and 24 h, then exposed to concentrations of AF, Au(NHC)Cl or $[Au(NHC)_2]PF_6$ (diluted in DMSO) equal to the corresponding 72 h-exposure IC_{50} values. A2780 control cells were treated with an equal concentration of DMSO. The final DMSO concentration in the growth media amounted to 0.1% of the total volume; this concentration of DMSO in A2780 culture has been reported neither to induce apoptosis nor to activate caspases. The incubation was stopped either at 6, 12 or 24 h; at the end of incubation 1–2 mL of medium was collected for metabolomic analyses. Cells were washed three times with PBS and then scraped in PBS supplemented with a protease and phosphatase inhibitor cocktail diluted in DMSO (Sigma-Aldrich). In agreement with the procedures for NMR sample preparation(*2–4*), cells were lysed by sonication in ice and then centrifuged at 200000g, 30 min, 4 °C. All the samples were stored at −80 °C until analysis.
For each condition, the samples from two different experiments based on three biological replicates were collected and analysed, for a total of six samples per group.
At the moment of the NMR analysis, frozen samples were thawed at room temperature and shaken before use. For cell lysates, 55 µL of ^2H_2O were added to 495 µL of each lysate sample. In the case of cell culture media, an aliquot of 300 µL of sodium phosphate buffer (70 mM Na_2HPO_4, 20% v/v ^2H_2O, 4.6 mM TMSP, pH was adjusted to the final value of 7.4 using 1 M HCl) was added to 300 µL of each medium sample. The mixtures were homogenized by vortexing for 30 s and transferred into 5 mm NMR tubes (Bruker BioSpin srl).

NMR experiments

The one-dimensional (1D) ^1H NMR spectra were recorded with a Bruker 600 MHz spectrometer (*5*, *6*), (Bruker BioSpin) optimized for metabolomic analysis, operating at 600.13 MHz proton Larmor frequency and equipped with a 5 mm PATXI ^1H–^{13}C–^{15}N and ^2H-decoupling probe including a z-axis gradient coil, an automatic tuning-matching (ATM) and an automatic refrigerated sample changer (SampleJet, Bruker BioSpin). A BTO 2000 thermocouple served for temperature stabilization at the level of approximately 0.1 K of the sample. Before measurement, to equilibrate temperature at 300 K, samples were kept for at least 5 minutes inside the NMR probe head.

The cell lysate samples were acquired with the Carr–Purcell– Meiboom–Gill (CPMG) sequence using a one-dimensional (1D) spin-echo sequence with water presaturation; 512 scans, 73728 data points, a spectral width of 12019 Hz and a relaxation delay of 4 s were used (Figure S1).

The growth media were acquired with a 1D nuclear Overhauser enhancement spectroscopy (NOESY)-presaturation pulse sequence; 64 scans, 98304 data points, a spectral width of 18028 Hz and a relaxation delay of 4 s were used (Figure S1).

The raw data were multiplied by a 0.5 Hz exponential line broadening before applying Fourier transform. Transformed spectra were automatically corrected for phase and baseline distortions and calibrated at the doublet of Ala at 1.49 ppm using TopSpin 3.6 (Bruker Biospin srl).

A two-dimensional (2D) ^{1}H-^{13}C Heteronuclear Single Quantum Coherence (HSQC; (hsqcetgpsisp2, Bruker)) spectrum in natural abundance was also acquired on cell lysates (Figure S2). A total of 80 scans were collected using: a spectral width of 12019.230 Hz for F2 and of 30178.449 Hz for F1; an acquisition time of 0.0852 s for F2 and 0.0021 s for F1; a relaxation delay of 2 s. The calibration of the spectra was performed to the doublet signal of alanine at 1.49 ppm (^{1}H chemical shift) and 19.03 ppm (^{13}C chemical shift).

1.5. Data analysis

The metabolites, whose peaks in the spectra were well resolved, were assigned and their levels analyzed using an in-house developed R script. We could identify and quantify 30 metabolites in the cell lysate spectra and 28 in the culture medium spectra (endo- and exo-metabolome in Figure. S1, respectively). The assignment was performed using an internal ^{1}H NMR spectral library of pure organic compounds (BBIOREFCODE, Bruker BioSpin srl), Chenomx software, public databases such as the Human Metabolome Database, stored reference NMR spectra of metabolites and literature data. Matching between new NMR data and databases was performed using the AMIX software (Bruker BioSpin srl). To further confirm the assignment of the most abundant and significant cell lysate metabolites and remove ambiguities, we used the natural abundance ^{1}H-^{13}C HSQC spectrum (Figure S2) and a spiking approach (Figure S3). The relative concentrations of the various metabolites were calculated by integrating the corresponding signals in the spectra using a home-made R script.

The nonparametric pairwise Wilcoxon–Mann–Whitney test was used to determine the meaningful metabolites; a p-value <0.05 was considered statistically significant. In order to reduce false discoveries, false discovery rate correction (FDR) was then applied using the Benjamini and Hochberg method. Log_2 Fold change (FC) was calculated for each metabolite to display how the metabolite levels vary upon the different comparison. FC is calculated as the median of the ratio of the metabolite concentrations in the spectra of the two paired samples (treated vs. control). In the growth media, metabolites were divided into two different classes,

i.e., those that are taken up from the medium and those that are released into the medium. For the molecules that are released, lower/higher concentration levels upon treatment correspond to a lower/higher release, while for the molecules that are taken up from the growth media, lower levels upon treatment correspond to a greater consumption of nutrients, i.e., increased uptake, and viceversa for higher levels.

PCA analysis was performed on binned NMR spectra, using R softaware. To this aim, each spectrum in the region 10.00–0.2 ppm was divided into 0.02 ppm chemical shift bins, and the corresponding spectral areas were integrated using the AMIX software. The area of each bin was normalized to the total spectral area, calculated with exclusion of the water and DMSO region (4.50–5.00 ppm and 2.90–2.60 ppm, respectively).

2. Results

Figures S4 and S5 show the main changes in the endo- and exo-metabolome, respectively, of A2780 cells following treatment with concentrations of Au(NHC)Cl and [Au(NHC)2]PF6 equal to the corresponding IC50 values at 72 h (Table S1), but for shorter treatment times (6, 12); for comparison purpose changes at 24 h treatment are also reported. Most of the changes at shorter times are relatively modest and the 24 h significant ones build-up quite slowly. Only glucose uptake and lactate, pyruvate and glycine release are significantly different for both compounds even after 6 h of treatment. At any of the measured times, [Au(NHC)2]PF6 produces the largest effects.

To better visualize the different effects caused by Auranofin, Au(NHC)Cl and [Au(NHC)2]PF6, the metabolic profiles of a representative triplicate of 24 h AF-treated cells (black) and respective controls (cyan) are compared with the cells treated with carbene-compounds for 24 h (red and green) and controls (blue) (Figure S6).

The controls all cluster in the same spatial region of the plot, while carbene-treated cells and AF-treated cells move into different areas, indicating the different changes observed in the cells after the treatment with the different Au(I)-drugs. Notably, [Au(NHC)2]PF6-treated cells are located farther from the controls than those treated with Au(NHC)Cl. This plot further confirms that Au(NHC)Cl and [Au(NHC)2]PF6 produce, on the whole, alterations in A2780 cells metabolism that are quite different from those caused by AF and that the second carbene compound is more effective that the other.

References

1. L. Messori, L. Marchetti, L. Massai, F. Scaletti, A. Guerri, I. Landini, S. Nobili, G. Perrone, E. Mini, P. Leoni, M. Pasquali, C. Gabbiani, Chemistry and Biology of Two Novel Gold(I) Carbene Complexes as Prospective Anticancer Agents. *Inorg. Chem.* **53**, 2396–2403 (2014).

2. V. Ghini, F. Magherini, L. Massai, L. Messori, P. Turano, Comparative NMR metabolomics of the responses of A2780 human ovarian cancer cells to clinically established Pt-based drugs. *Dalton Trans.* **51**, 12512–12523 (2022).

3. V. Ghini, T. Senzacqua, L. Massai, T. Gamberi, L. Messori, P. Turano, NMR reveals the metabolic changes induced by auranofin in A2780 cancer cells: evidence for glutathione dysregulation. *Dalton Trans.* **50**, 6349–6355 (2021).

4. C. Bernacchioni, V. Ghini, F. Cencetti, L. Japtok, C. Donati, P. Bruni, P. Turano, NMR metabolomics highlights sphingosine kinase-1 as a new molecular switch in the orchestration of aberrant metabolic phenotype in cancer cells. *Mol. Oncol.* **11**, 517–533 (2017).

5. A. Vignoli, V. Ghini, G. Meoni, C. Licari, P. G. Takis, L. Tenori, P. Turano, C. Luchinat, High-Throughput Metabolomics by 1D NMR. *Angew. Chem. Int. Ed.* **58**, 968–994 (2019).

6. P. G. Takis, V. Ghini, L. Tenori, P. Turano, C. Luchinat, Uniqueness of the NMR approach to metabolomics. *TrAC Trends Anal. Chem.* **120**, 115300 (2019).

3. Supplementary Tables

Table S1. IC_{50} values (μM) in A2780 cells, obtained through an MTT assay using an exposure time of 72 h.

**IC_{50} at 72 h**	**A2780**
**AF**	0.7 ± 0.05 μM
**Au(NHC)Cl**	3.5± 0.91 μM
**$[Au(NHC)_2]PF_6$**	0.1 ± 0.01 μM

4. Supplementary Figures

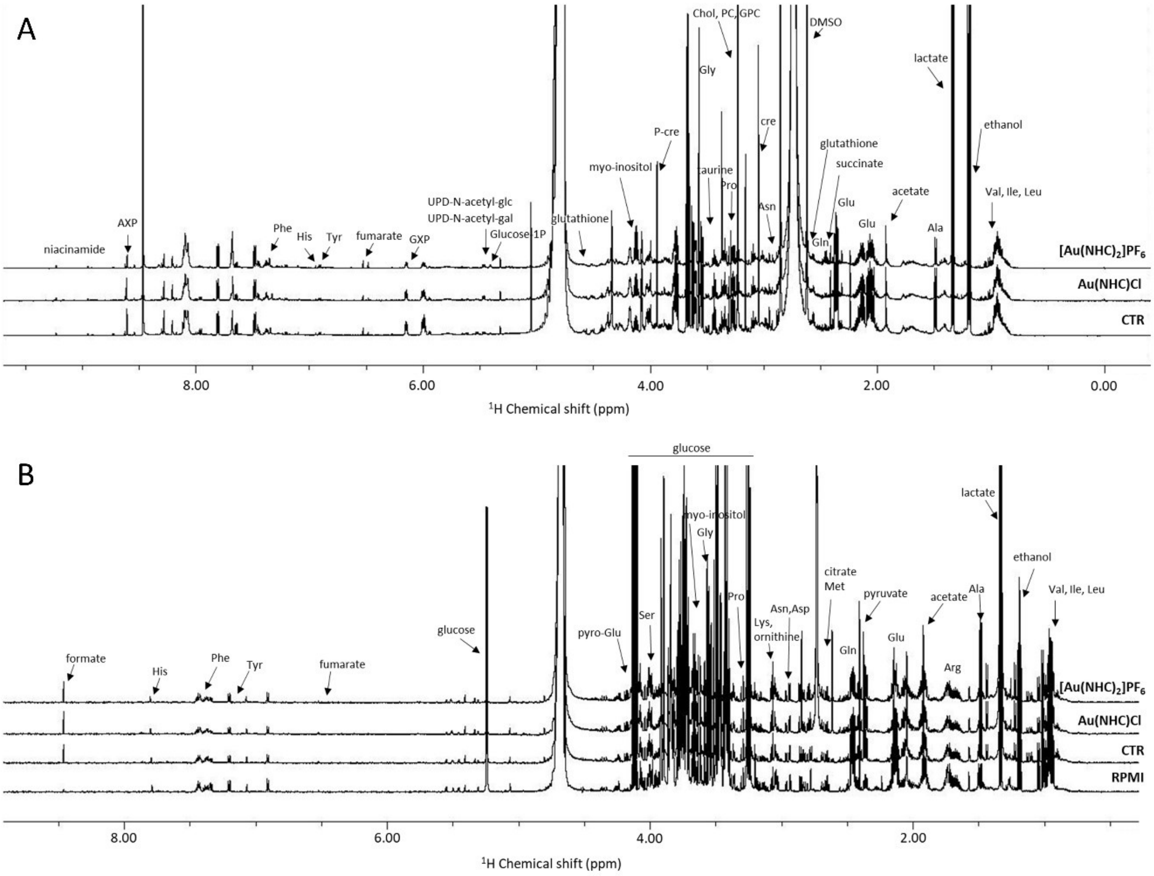

Figure S1. Representative ^{1}H NMR spectra of 24 h treated and control A2780 cells: A) CPMG of cell lysates (endo-metabolome); B) NOESY of the corresponding growth media (exo-metabolome); the spectrum of fresh supplemented medium is reported for comparison. The assignment of the main peaks is reported.

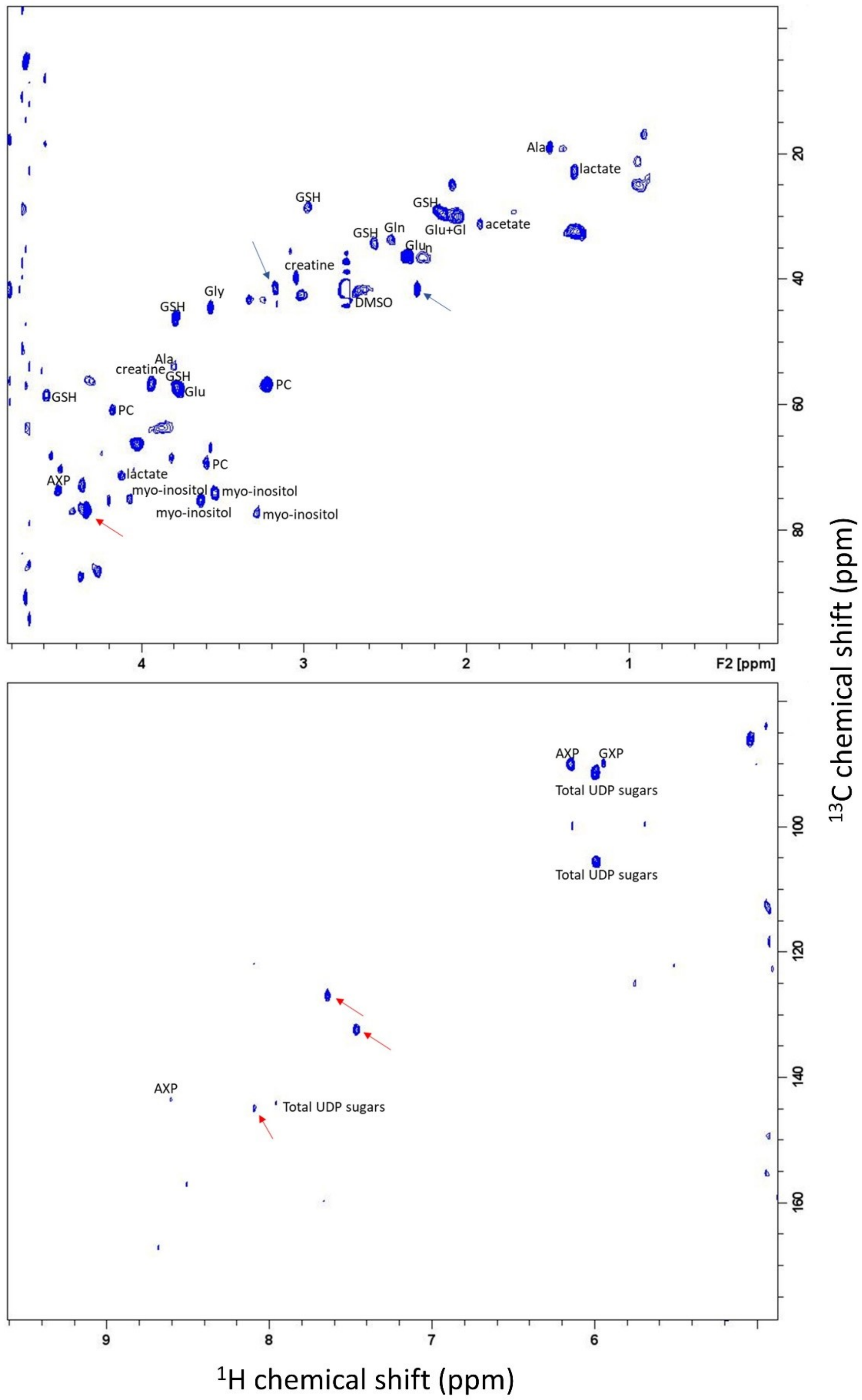

Figure S2. ^{1}H-^{13}C HSQC spectrum of a representative cell lysate sample. Top panel: aliphatic region; bottom panel: aromatic region. The assignment of the main peaks is reported. Red arrows indicate signals arising from the protease and phosphatase inhibitor cocktail used to

quench the cellular metabolism at the moment of cell lysate sample preparation. Satellite signals of the intense resonance of DMSO are indicated by blue arrows.

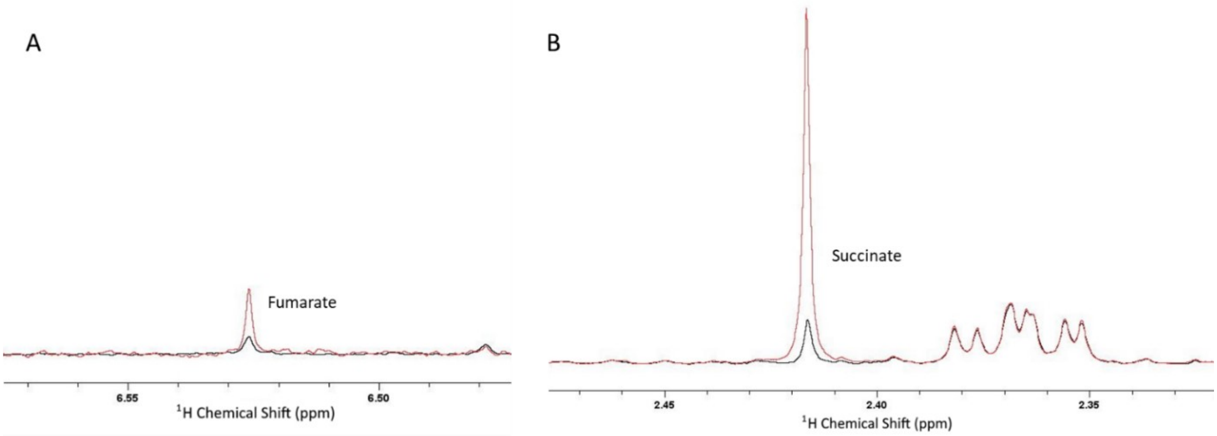

Figure S3. ^{1}H CPMG spectrum of a representative cell lysate sample before (black traces) and after (red traces) spiking of A) fumarate and B) succinate.

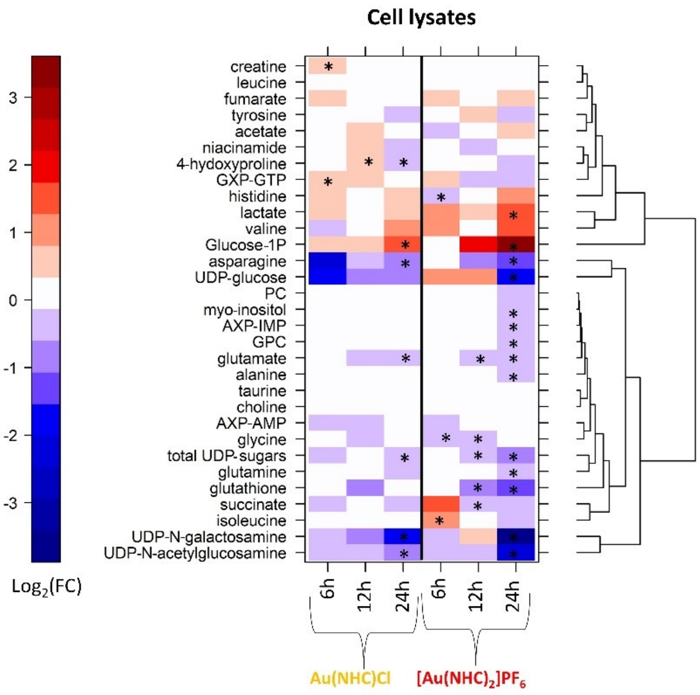

Figure S4. Level plot of fold changes of the intracellular metabolites (cell lysates) upon 6, 12 and 24 h of treatment of A2780 cells with Au(NHC)Cl and [Au(NHC)2]PF6. Red/blue colours indicate the higher/lower metabolite concentration in Au(I) drug-treated cells with respect to control samples ($\log_2$(FC)). The brightness of the colours corresponds to the magnitude of FC. Asterisks indicate statistical significance (p-value <0.05).

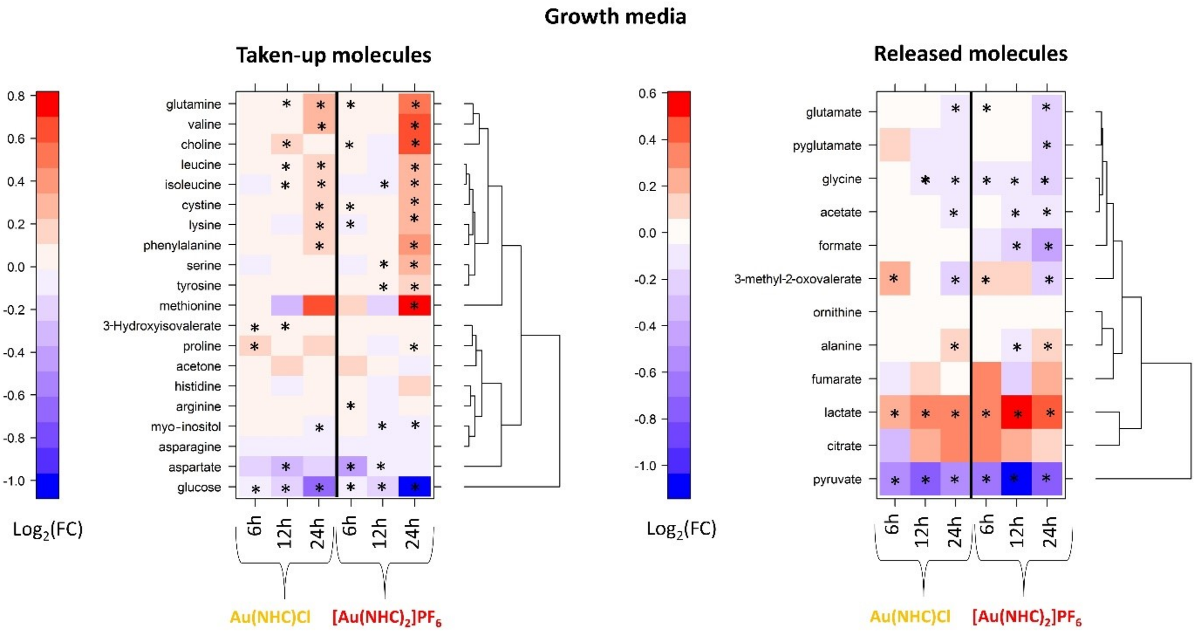

Figure S5. Level plots of fold changes of the extracellular metabolites (growth media) upon 6, 12 and 24 h of treatment of A2780 cells with Au(NHC)Cl and [Au(NHC)$_2$]PF$_6$. Red/blue colours indicate the higher/lower metabolite concentration in Au(I) drug-treated cells with respect to control samples (log$_2$(FC)). The metabolites are divided into two different classes, i.e. those that are taken up from the medium and those that are released into the medium. While for the molecules that are released, lower concentration levels upon treatment mean a lower release, for the molecules that are taken up from the starting media, lower levels upon treatment mean a greater consumption of nutrients, i.e. increased uptake, and viceversa. The brightness of the colours corresponds to the magnitude of FC. Asterisks indicate statistical significance (p-value <0.05).

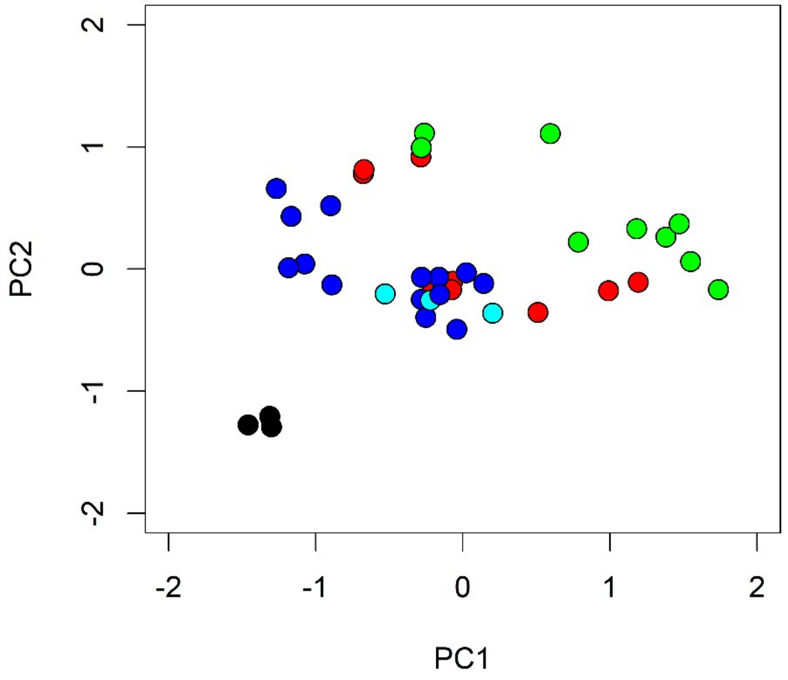

Figure S6. PCA-CA score plot of A2780 cells lysates. Each dot represents a different NMR sample. 24 h AF-treated cells and their controls are shown in black and cyan, respectively. 24 h carbene-treated cells and their controls are shown in red (Au(NHC)Cl), green ([Au(NHC)$_2$]PF$_6$) and blue (controls).
